# Prediction Beyond the Borders: ERP Indices of Boundary Extension-Related Error

**DOI:** 10.1371/journal.pone.0074245

**Published:** 2013-09-12

**Authors:** István Czigler, Helene Intraub, Gábor Stefanics

**Affiliations:** 1 Institute of Cognitive Neuroscience and Psychology, Research Center for Natural Sciences, Hungarian Academy of Sciences, Budapest, Hungary; 2 Department of Psychology, University of Delaware, Newark, Delaware, United States of America; 3 Translational Neuromodeling Unit (TNU), Institute for Biomedical Engineering, University of Zurich & ETH Zurich, Zurich, Switzerland; 4 Laboratory for Social and Neural Systems Research, Department of Economics, University of Zurich, Zurich, Switzerland; University of Surrey, United Kingdom

## Abstract

Boundary extension (BE) is a rapidly occurring memory error in which participants incorrectly remember having seen beyond the boundaries of a view. However, behavioral data has provided no insight into how quickly after the onset of a test picture the effect is detected. To determine the time course of BE from neural responses we conducted a BE experiment while recording EEG. We exploited a diagnostic response asymmetry to mismatched views (a closer and wider view of the same scene) in which the same pair of views is rated as more similar when the closer item is shown first than vice versa. On each trial, a closer or wider view was presented for 250 ms followed by a 250-ms mask and either the identical view or a mismatched view. Boundary ratings replicated the typical asymmetry. We found a similar asymmetry in ERP responses in the 265-285 ms interval where the second member of the close-then-wide pairs evoked less negative responses at left parieto-temporal sites compared to the wide-then-close condition. We also found diagnostic ERP effects in the 500-560 ms range, where ERPs to wide-then-close pairs were more positive at centro-parietal sites than in the other three conditions, which is thought to be related to participants’ confidence in their perceptual decision. The ERP effect in the 265-285 ms range suggests the falsely remembered region beyond the view-boundaries of S1 is rapidly available and impacts assessment of the test picture within the first 265 ms of viewing, suggesting that extrapolated scene structure may be computed rapidly enough to play a role in the integration of successive views during visual scanning.

## Introduction

**Figure 1:**
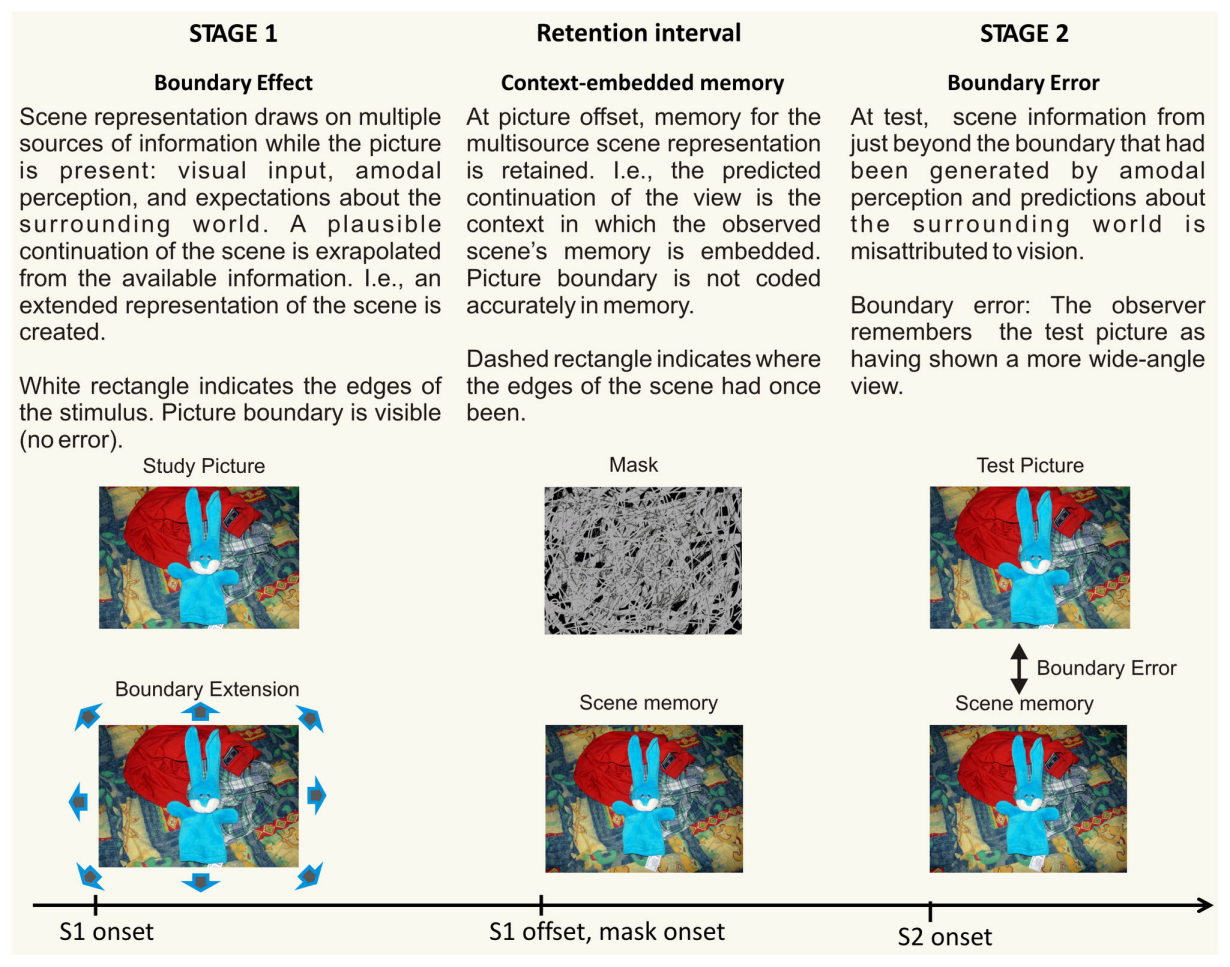
A schematic model of the boundary extension process in the framework of an S1-S2 paradigm.

When remembering a view of a scene, observers frequently remember having seen more of the world than was shown (boundary extension [1]). This is thought to reflect the rapid computation of expected surrounding space during scene perception. The neural underpinnings of boundary extension are just beginning to be explored. The process of boundary extension is thought to involve two stages [2,3]. In the first stage, after the presentation of a scene, the plausible continuation of the viewed scene is computed, i.e. the representation of the scene is extrapolated beyond its original boundaries. In the second stage, at test, participants accept some of this extrapolated continuation as having been seen. What type of process might be taking place at test? Figure 1 illustrates one theoretical account of the processes that may lead to this rapidly occurring error of commission [3,4]. During Stage 1, at encoding, a scene representation is constructed that goes beyond the visual information available in the picture by drawing on multiple top-down sources of information (e.g., amodal perception and general scene knowledge). This “scene extrapolation” can be thought of as an *automatic predictive process* that places the visible information into it larger likely context. While the picture is visible (Stage 1 in the figure), the boundary error does not occur because the participant can see where the boundaries are. The dividing line between the vision input and amodal continuation of objects and surfaces cropped by the boundary is clearly discernible.

In Stage 2, however, when the sensory information (picture) is no longer present, the entire scene representation is maintained in memory. The original sources of the information are not “tagged”, leading to a fuzzy boundary between information that was originally inside the boundary and extrapolated information that is just beyond the boundary. The original boundaries are no longer easy to discern. At retrieval in Stage 2 when the test picture appears, the participants must make a comparison between what they see now and what they remember haven seen before which is now a “boundary-extended” version of S1. The erroneous acceptance of non-visually generated, i.e., extrapolated, information to vision leads to the empirical outcome that is termed boundary extension. The participant remembers having seen beyond the boundaries of the view.

A recent neuroimaging study [2] investigated the first phase of the process of boundary extension. In this elegant study S1 trials were sorted according to the corresponding behavioral responses to S2 scenes. Analysis of trials with behavioral responses diagnostic to BE indicated that extrapolation of scenes during first viewing (S1) was associated with increased activity in the hippocampus (HC) and parahippocampal cortex (PHC), with the HC driving the BE effect by exerting top-down control on PHC and the visual cortex. Another recent fMRI study, where neural adaptation effects were examined in a BE paradigm [5], has suggested that boundary extension is associated with neural activity in the parahippocampal place area (PPA) and retrosplenial cortex (RSC). A study of boundary extension in patients with bilateral hippocampal lesions has suggested possible hippocampal involvement [6]. Here we sought to determine how quickly after the onset of a test picture boundary extension can be detected by measuring even-related potentials (ERPs) in the context of a brief-presentation boundary extension task.

Boundary extension occurs rapidly. It has been observed across retention intervals that simulate a saccade (e.g. 42 ms) and across actual saccades [4,7]. In these experiments, on each trial the observers see a briefly presented picture interrupted by a brief mask followed by the test picture (either the identical view or a close or more wide-angled view). The participant then rates the test picture on a 5-point scale as being the same, or closer-up or more wide-angled. This behavioral task revealed that a brief interruption was sufficient to elicit boundary extension, but cannot provide insight into when between test-picture onset and the participant’s behavioral response the impact of the error first occurs. Is it rapid, or does it require many seconds to unfold?

A similar problem was posed by behavioral studies exploring the early time course of scene categorization (“gist” acquisition). Behavioral research demonstrated that very brief presentations (e.g., 100 ms [8]) were sufficient to elicit categorization, but when between picture onset and the behavioral response categorization had actually taken place was unclear. To provide a tighter time window than available in behavioral research alone to answer this question, Thorpe, Fize, & Marlot [9] measured event-related potentials (ERPs) to rapid serial visual presentation (RSVP) sequences when the sequence either did or did not include a category change. Their ERPs suggested that scene identification occurred at least as soon as 150 ms following picture onset. We sought to use ERP to gain insight into the speed with which boundary extension is detected at test.

To accomplish this we measured ERPs to S2 scenes in the context of a boundary extension task. The importance of understanding the time course of the second stage of the boundary extension phenomenon stems from the possibility that it may play a role in our ability to perceive a continuous, surrounding world based upon successive, discrete eye fixations [10,11]. Boundary extension is a memory error (a “memory illusion”, [12]) that may have an adaptive function in anticipating the continuity of layout across successive views. In support of this, Gottesman [13] has demonstrated that boundary extension can prime perception of the region surrounding a given view, when the region is actually presented later in the experimental session. Boundary extension appears to be specifically related to scene perception, in that it occurs in memory for views of objects in scenes but not for the same objects when presented on blank backgrounds [14,15].

In the present research, we chose to exploit a well-established response asymmetry in memory for the boundaries of mismatched views (see 16,17 for reviews). Given a pair of photographs, one a closer view and one of more wide-angle view of the same scene, when the closer view is shown first and the wider view is shown at test (CW), the difference between the two is rated as being relatively small in comparison to trials on which the wider view is shown first and the closer view is shown at test (WC). The explanation of this effect is that boundary extension in memory of the first view (people remember it as being more wide-angle) would minimize the difference between the stimulus and test picture in the case of CW, and maximize it in the case of WC. The major goal was to determine if an asymmetrical pattern in the ERP signature is associated with these two types of trials, and if so, what the timing of this discrepant response would be.

We were encouraged by fMRI research [5], in which this asymmetry was observed in the neural responses of selective areas of the brain. In that study participants simply viewed a series of photographs shown for a few seconds each. Sometimes a closer view was followed by the wider view (CW) or vice versa (WC) and sometimes the identical close or wide picture was simply repeated (CC and WW, respectively). Repetition attenuation (see e.g. 18, for a review) of the neural response in the PPA and RSC occurred when closer views were followed by wider views (CW trials), signifying that these different views were treated as similar; but no repetition attenuation occurred when the wider views were followed by closer views (WC trials), suggesting that the views were treated as being different. Both brain areas (PPA and RSC) are thought to be scene-selective areas [19,20].

In contrast, at the same time, no asymmetry was observed in the neural response in lateral occipital cortex (LOC), a region associated with object recognition. Here repetition attenuation occurred in both the WC and CW conditions, signifying recognition of the same object in spite of the difference in size conveyed by the closer and wider views. Finally, this fMRI research [5] demonstrated that the response asymmetry occurred when these participants took part in a boundary extension task in which participants explicitly rated the test picture using a typical boundary rating task: pictures on CW trials were rated as being more similar than were pictures on WC trials.

In the present ERP study, unlike the fMRI study, stimulus presentation was brief, since EEG reflects neural processing with ms precision. We chose a stimulus duration of 250 ms because it has been shown to elicit boundary extension under a variety of conditions [4,21,22]. The specific purpose of our experiment was to determine if the ERP signature would mirror the behavioral response asymmetry, and if so, how soon after onset of the test picture this asymmetry was in evidence. We hypothesized that activity change in a posterior location might indicate an automatic match-mismatch distinction in the visual system (e.g., [23,24,25]).

The presentation sequence in our study included the critical CW and WC stimulus-test pairs, as well as CC and WW stimulus-test pairs. Participants rated the second member of a pair as being the same, more close-up, or more wide-angled than the first picture on a 5-point Likert scale centered on “same (0)”. Although this is a common test procedure in the boundary extension literature, what was uncommon about the present experiment is that ERP research required hundreds of trials. In research on boundary extension, the procedure involves memory for a relatively small number of trials (usually fewer than 36). This is because picture memory is prone not only to boundary extension but to other types of errors as well that can interact with this unidirectional error.

For example, the report [26] of greater boundary extension when memory was tested immediately following an 18 picture sequence as compared to a 2-day delay, is paradoxical (suggesting better memory after a long delay) until one recognizes that a second memory error across pictures (regression to the mean view), which increased over time was also impacting memory. This leads to what was referred to as the dual-component view of boundary extension (see 16,17 for reviews). Subsequently other errors (e.g., a downward shift in object placement [27]), has led to the recognition that there are likely several memory factors than can interact with boundary extension over time. We were concerned that the relatively large number of trials required in the ERP study would elicit other problems in memory that would interact with detection of boundary extension. For example, proactive interference caused by rating many single-object pictures across large numbers of trials. However, we thought that the response asymmetry to CW and WC pairs might be strong enough to survive hundreds of trials.

The current study is the first to investigate BE-related ERP effects; hence in lack of a priori knowledge of possible effect locations or latencies we applied a mass univariate analyses [28]. The advantage of this method is that by applying a large number of univariate tests (t-tests) ERPs can be compared exhaustively at many channels and time points. This benefit though comes at the cost that mass univariate analyses are more conservative and less likely to detect effects than conventional statistical tests.

We assumed that BE occurs after the presentation of every stimuli, that is, during encoding of every image a distorted memory is created [4,7]. However, this process can only be studied by means of secondary test items with different viewing angles than the original images. Assuming that the behavioral asymmetry to CW and WC pairs is obtained, this could be reflected in differences in the ERPs to the test stimuli. Specifically, we expected more negative ERPs to the test picture on WC trials (i.e., the closer) than ERPs to the test pictures on the CW trials (i.e., the wider). This pattern of results would suggest that the bigger “mismatch” between pictures was on the WC trials.

Stimuli requiring attention and behavioral response during a perceptual task usually elicit a late positive event-related potential, the P3b ( [29,30,31]). Assuming that the behavioral asymmetry is obtained, this could be reflected in the P3b component in two different ways. In one scenario, P3b to the test picture on CW trials (i.e., the W) would differ from ERPs to the test pictures on the other three types of trials (WC, CC, and WW). This pattern would suggest that on CW trials, the test picture more closely “matches” the first picture in the pair than on any of the other trials. In the other scenario, P3b to the test picture on the WC trials (i.e., the C) would differ from ERPs to the test picture on the other three types of trials (CW, CC, and WW). This pattern would suggest that the greatest “mismatch” between pictures was on the WC trials.

## Methods

### Ethics Statement

The experimental procedures were approved by the Joint Psychological Research Ethics Committee of the Hungarian Psychology Faculties and Research Labs, Budapest, Hungary. Written informed consent was obtained from all participants after the procedures were explained. The experiments were conducted in full compliance with the Declaration of Helsinki.

### Participants

Participants were 23 students recruited from various universities in Budapest who were paid for their participation (11 female and 12 male, *M* = 21.8, *SD* = 2.24). They had normal or corrected-to-normal vision. Due to abundant alpha activity or low trial number caused by excessive artifacts (eye blinks, movements, etc.), data of 4 additional participants were excluded from the analysis.

### Stimuli

The stimuli were 256 pairs of color photographs, showing a closer and a wider view of the same scene. Closer view stimuli were created by “zooming in” to the center of the wider view images by cropping 15% of the images. Each scene included a single object or object cluster on a natural background. Each picture subtended 18.7° visual angle horizontally and 15.8° vertically. Stimuli were presented on a dark grey background. A black fixation cross was presented in the center of the screen in a dark grey disk subtending 1.17° visual angle. To minimize eye-movements subjects were instructed to fixate at the cross throughout the experiment. The image set partly consisted of scenes that had been used in a prior study [5], and scenes that were created by the authors and taken from the internet. The stimulus set is available upon request from the authors for research purposes.

### Design and Procedure

**Figure 2:**
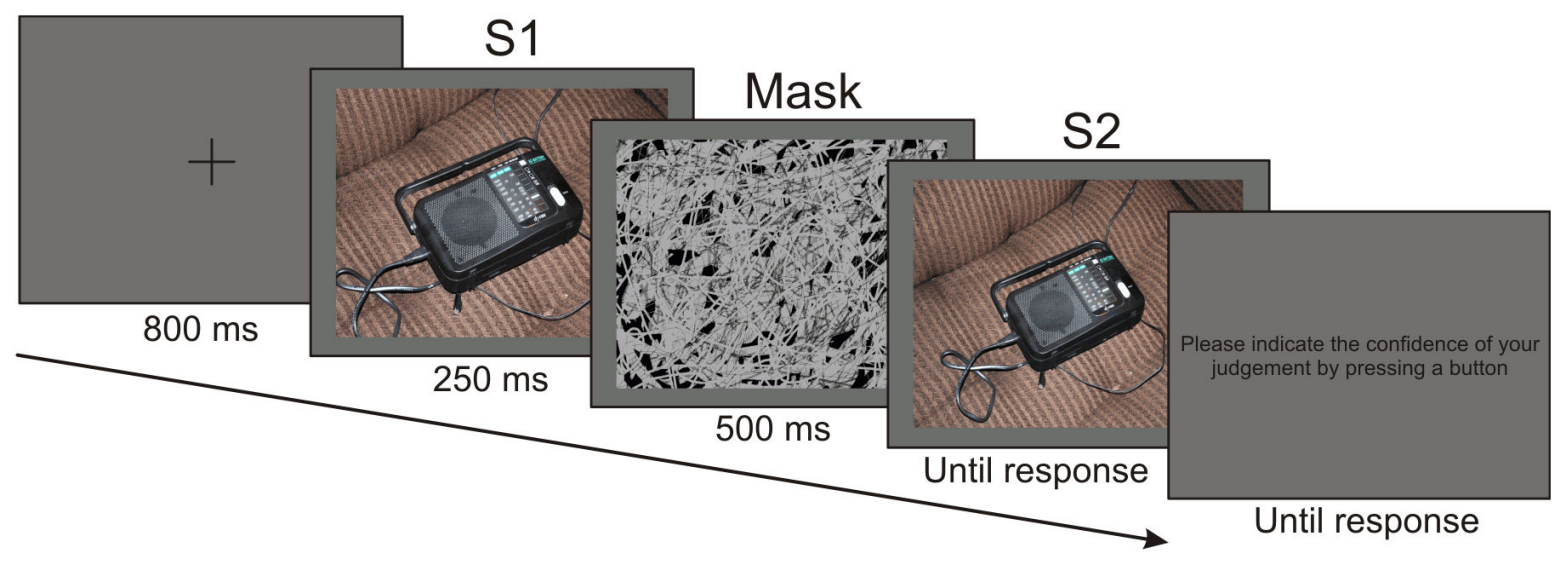
Schematic illustration of an experimental trial showing an example of a close-wide (CW) pair. Event durations are indicated under each screen.

Each participant saw a total of 512 trials, a sample trial is shown in Figure 2. On half the trials the stimulus was the closer view and on half the wider view, randomly intermixed. Each block included 64 stimulus-test pairs: 16 close-close (CC), 16 wide-wide (WW), 16 close-wide (CW) and 16 wide-close (WC). Assignment of the 256 individual scenes into CC, WW, CW and WC pairs were randomized for each participant. Each trial began with a presentation of a black fixation cross for 800 ms on a dark-grey background, which was followed by the first member of the stimulus pair (S1) for 250 ms. A visual noise mask was presented immediately after the S1 for 500 ms. This was followed by the second member of the pair (S2) presented on the screen until participants pressed a button indicating their perceptual scaling judgments. The instruction was to rate the S2 scene relative to S1 on a five-step scale (1. ‘Much closer’, 2. ‘Closer’, 3. ‘Same’, 4. ‘Farther’, 5. ‘Much farther’). It was explained that when the camera was ‘farther away’, the object would be smaller and the more of the background would be visible. A response box was provided with five buttons arranged in a horizontal row. The leftmost button was to be pressed to indicate ‘Much closer’ rating, and the rightmost button for the ‘Much farther’ rating. In half of the blocks, participants pressed the buttons with the left hand, in the other half with the right hand. Response speed was not emphasized and the S2 scene was presented until button press. After this rating, the S2 was replaced on the screen by the instruction to participants to indicate their confidence on a five-point Likert scale (1. ‘Not sure at all’, 2. ‘Not sure’, 3. ‘Maybe’, 4. ‘Sure’, 5. ‘Absolutely sure’) by pressing a button. Subjects were instructed to use the buttons of the response box to indicate their confidence. The leftmost button was to be pressed to indicate lowest confidence (‘Not sure at all’), and the rightmost button for the highest confidence (‘Absolutely sure’). At the beginning of the experiment a short training block was presented to ensure that participants understood the task. Camera angle judgment response time was analyzed with two-way analysis of variance (ANOVA) with factors camera angle (closer vs. same vs. wider) × type (CC vs. WW vs. CW vs. WC), whereas confidence rating response time was analyzed with ANOVA with factors level (high vs. medium vs. low) × type (CC vs. WW vs. CW vs. WC). Due to low frequency of responses in the categories of ‘Much closer’ and ‘Much farther’ for camera angle, and ‘Not sure at all’ for confidence ratings, responses within the 1-2 and 4-5 response categories were collapsed. Greenhouse–Geisser correction of the degrees of freedom was applied where appropriate. Significant interactions were further specified by Tukey’s Honestly Significant Difference (HSD) post-hoc tests. Reported results were significant to at least the p<.05 level. At the beginning of the experiment a short training block was presented to ensure that participants understood the task.

### EEG recording and pre-processing

EEG was recorded from 63 Ag/AgCl electrodes (AF7, Fp1, Fpz, Fp2, AF8, AF3, AFz, AF4, F7, F5, F3, F1, FZ, F2, F4, F6, F8, FT7, FC5, FC3, FC1, FCz, FC2, FC4, FC6, FT8, T7, C5, C3, C1, Cz, C2, C4, C6, T8, A1, TP7, CP5, CP3, CP1, CPz, CP2, CP4, CP6, TP8, A2, P7, P5, P3, P1, Pz, P2, P4, P6, P8, PO7, PO3, POz, PO4, PO8, O1, Oz, O2) covering the whole scalp according to a modified international 10–20 system. An electrode attached to the tip of the nose was used as reference. The ground electrode was placed on the forehead. EEG was recorded from DC with a low-pass filter at 100 Hz. Eye movements were monitored by two horizontal and two vertical bipolar EOG electrodes. Data were digitized at 32 bit resolution and a sampling rate of 500 Hz (Neuroscan Synamp). EEG was filtered off-line between 0.1 and 30 Hz (24 dB/octave) and re-referenced to the common average. All subsequent data analyses were carried out off-line on PC using built-in and self-developed functions as well as the freeware EEGLAB toolbox [32] in Matlab (MathWorks, Natick, MA).

### Analyses and comparisons

For all test stimulus types epochs of 1000 ms including a 100 ms pre-stimulus period were extracted from the continuous EEG for further analysis. Epochs were baseline-corrected for the -100-0 ms period and averaged separately for the CC, WW, CW and WC trials. To avoid potential artifacts, epochs with potential values exceeding ±75 μV on any EEG or EOG channel were rejected from the analysis.

A data-driven statistical approach was applied to reveal reliable differences between WC and CW ERPs. ERPs from these conditions to S2 stimuli were submitted to a repeated measures, two-tailed permutation test based on the tmax statistic [33] using a family-wise alpha level of 0.05. To increase the statistical power of our analysis data was downsampled to 100 Hz and thirty-four channels out of the original 61 providing even full head-coverage were selected [28].

All time points between 100 and 600 ms at 34 scalp electrodes were included in the test (i.e., 1734 total comparisons). 2500 random within-subject permutations of the data were used to estimate the distribution of the null hypothesis (i.e., no difference between conditions). Based on this estimate, critical *t*-scores of +/- 4.7419 (*df* = 22) were derived, corresponding to a test-wise alpha level of 0.000099. In other words, any differences in the original data that exceeded a *t*-score of +/-4.7419 were deemed reliable.

A similar statistical approach was used to compare ERPs to S2 stimuli from the CC and WW conditions where no difference was expected. Since S2 stimuli in both conditions would differ equally from the distorted memory, mismatch signals with equal magnitude should be present in responses to S2.

Visual inspection of the ERPs to S2 stimuli revealed a prominent positive response in the 500-560 ms interval which was identified as P3b component. Based on the well-known topographic distribution of the P3b with parietal maximum, three Regions of Interest (ROIs) were defined to test for possible differences between all four conditions. Electrodes CP1 and P1 were selected for the left, CPz and Pz for the midline and CP2, P2 for the right ROI. Mean amplitude values of this latency range to S2 stimuli were submitted to a two-way analysis of variance (ANOVA) with factors type (CC vs. WW vs. CW vs. WC) × laterality (left vs. middle vs. right). Greenhouse–Geisser correction of the degrees of freedom was applied where appropriate. Significant interactions were further specified by Tukey’s Honestly Significant Difference (HSD) post-hoc tests. Reported results were significant to at least the *p*<.05 level.

## Results

### Behavioral results

**Figure 3:**
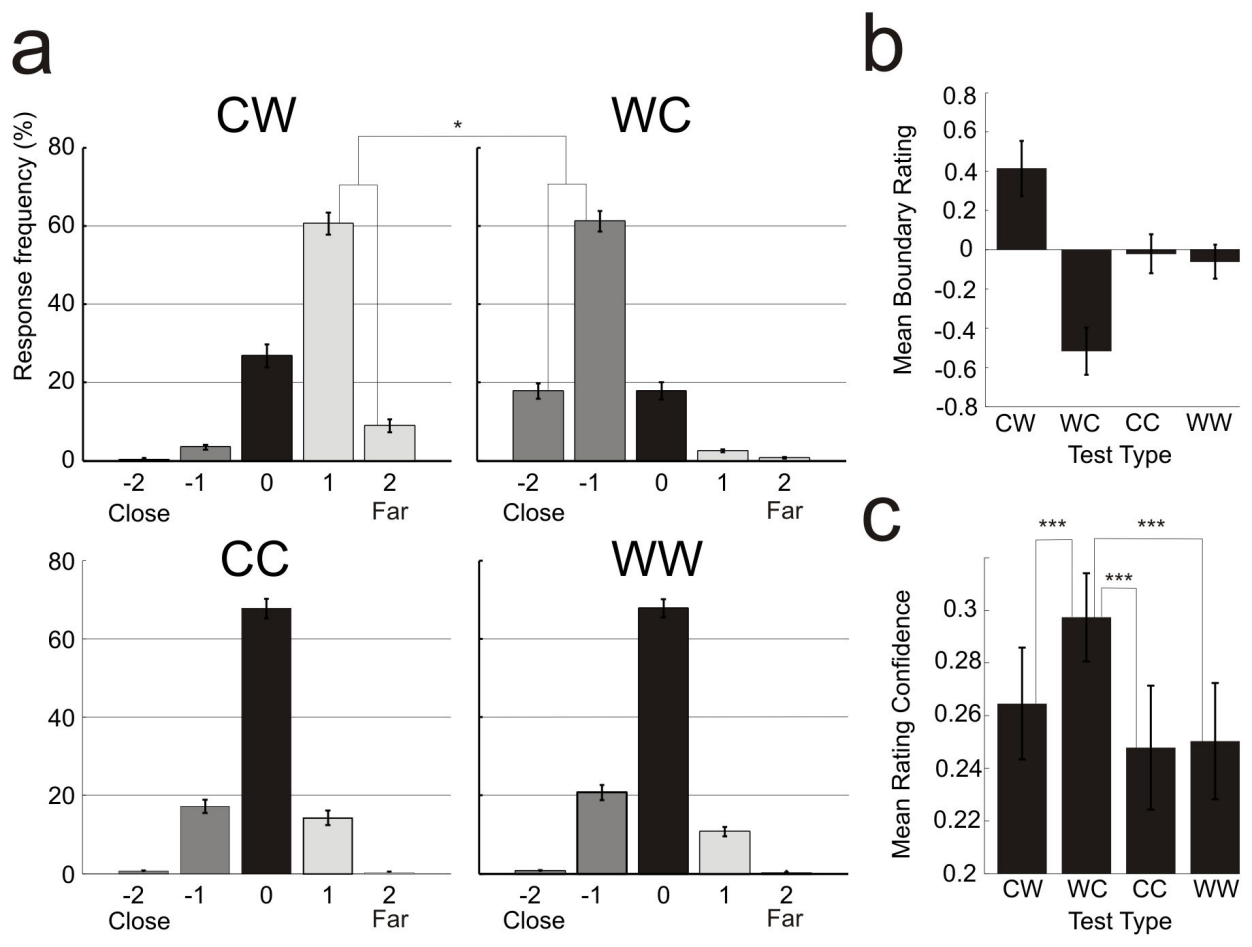
Frequency of closer/wider ratings (a). Mean boundary ratings across all blocks (b). Confidence ratings (c). Significant differences are indicated by asterisks (*p<0.05, ***p<0.001). Whiskers indicate standard error mean (SEM).

Figure 3 shows the frequency of each rating on the 5-point boundary scale, the mean boundary rating for each trial type, and the mean confidence rating for each trial type. BE is demonstrated by two diagnostics. The first is that the second member of identical pairs should be judged as closer than the first member, especially in case of the CC pairs. According to the results of t-tests, ratings on the CC pairs were not different from zero [*t*(22) = 1.93, *p* >.05], while the t-value on WW was significant [*t*(22) = 7.87, *p* < .01]. Given the initial concern that large numbers of trials would minimize or eliminate BE, we analyzed the first block separately [note that even in a single block the number of trials (64) was larger than in the previous BE studies (36 trials e.g., in Intraub and Dickinson, 2008)]. T-tests of mean boundary ratings showed that both the CC [*t*(22) = 2.48, *p* < .05, d = 0.57] and WW [*t*(22) = 3.80, *p* < .001, d = 0.85] trials yielded significant BE [the mean ratings differed from “same (0)”], and Cohen’s *d* measures of effect sizes indicate that effects for CC and WW are medium and large, respectively.

The second diagnostic of BE, was the one that was most critical for our research. It is the response asymmetry to mismatched pairs. Here the expectation is for a larger difference from zero on WC trials than on CW trials. To investigate the appearance of this difference, WC ratings were multiplied by -1, and these values were compared to the CW ratings in a paired *t*-test. As expected, the magnitude of the ‘farther’ (CW) ratings was significantly smaller than that of ‘closer’ (WC) ratings [*t*(22) = 3.12, *p* < .05, d = 5.12] indicating that subjects systematically underestimated changes in scope of the view for close-wide relative to wide-close pairs. Thus in spite of the large number of trials, the response asymmetry was present in the behavioral data.

Figure 3b shows confidence ratings in the four types of pairs which were compared in a single factor ANOVA. The significant difference [*F*(3,66) = 10.20, ε = 0.64, *p* < .001, *η* ^2^= 0.32] and the results of the Tukey HSD tests show, that participants were more confident at the WC pairs than in the other three type of pairs.

Analysis of camera angle judgment response time yielded a significant main effect of camera angle (*F*(2, 28)=15.07, ε = 0.96, *p*<.0001, *η2*=0.52), which was caused by faster ‘same’ responses (1303 ms, *sd*=118) than ‘closer’ (1666 ms, *sd*=149) and ‘wider’ (1628 ms, *sd*=131) responses. A significant camera angle × type interaction was observed (*F*(6, 84)=3.03, ε = 0.44, *p*<.05, *η2*=0.18), which was caused by faster ‘same’ (1162 ms, *sd*=81) than ‘wider’ (1671 ms, *sd*=145) responses in the CC condition (*p*<.05), faster ‘same’ (1129 ms, *sd*=77) than ‘wider’ (1672 ms, *sd*=149) responses in the WW condition (*p*<.05), and faster ‘same’ (1440 ms, *sd*=168) than ‘closer’ (1911 ms, *sd*=236) responses in the CW condition (*p*<.05).

Analysis of confidence rating response time yielded a marginally significant main effect of level (*F*(2, 16)=4.3870, ε = 0.59, p<.06, *η2*=0.35), which was caused by faster high-confidence (734 ms, *sd*=103) than low-confidence (1100 ms, *sd*=150) responses (*p*<.05). No other effects or interaction reached significance.

### Event-related potentials

Brain electrical activity was markedly different at the posterior and anterior regions. Posterior ERPs were dominated by the P1 (~120 ms), N1 (~170 ms), the P2 (~250 ms) and the P3b components (~500 ms), whereas ERPs at the anterior locations consisted of a negativity (~120 ms), followed by the P2 and the late positivity. The P3b was largest over centro-parietal locations.

**Figure 4:**
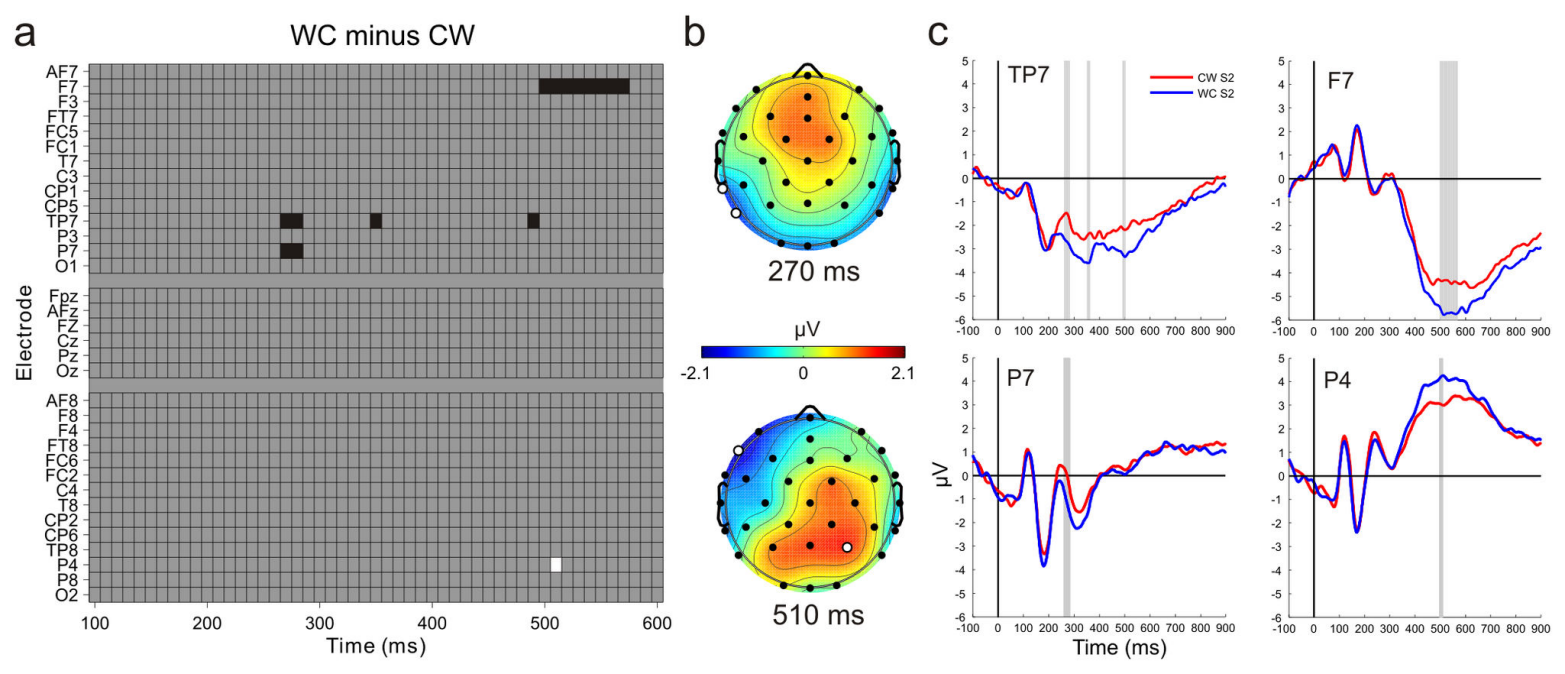
Results of mass univariate statistical analysis. (a) Each box of the raster diagram represents the results of a t-test. Black boxes indicate that the WC minus CW difference wave was significantly positive at that time point and electrode, white indicates significantly positive differences, after effectively correcting for multiple comparisons). (b) Topographic maps of WC minus CW difference potentials at timepoints within intervals where significant differences were observed. Dots mark electrodes used for mass univariate tests. White dots mark significant electrodes. (c) S2 ERPs in the WC and CW conditions. Gray shadings indicate timepoints where significant differences were found.

The raster diagram in Figure 4 shows the significant electrode-timepoints, corresponding topographic maps and ERPs. Mass univariate analysis (tmax statistic [33]) of the WC minus CW difference revealed significantly more negative ERP responses to WC compared to CW test stimuli at the 265-285 ms timepoints at left posterior electrodes (TP7, P7). A similar effect was observed at two following timepoints at 345-350 ms, and 285-295 ms at the TP7 electrode. In a later time window corresponding to the P3b response, a more positive ERP was found for the WC compared to the CW test stimuli at the 505-515 ms timepoints at the P4 electrode, and correspondingly a more negative response was observed for the WC compared to the CW test stimuli at the 495-575 ms time points at a left frontal site (F7). The mass univariate statistical analysis similar to that described above failed to reveal any statistically significant differences between ERPs to S2 stimuli in the CC and WW conditions. Although by not rejecting the null hypotheses (i.e. that S2 ERPs in CC and WW differ) this result only indirectly supports the idea that S2 stimuli do not differ in the CC and WW conditions, and that no systematic ERP differences were present due to potential asymmetric response expectancies based on S1. Nevertheless it also indicates that the mass univariate statistics applied here is a reliable method and significant effects which survive the correction for multiple comparisons reflect robust differences between conditions.

**Figure 5:**
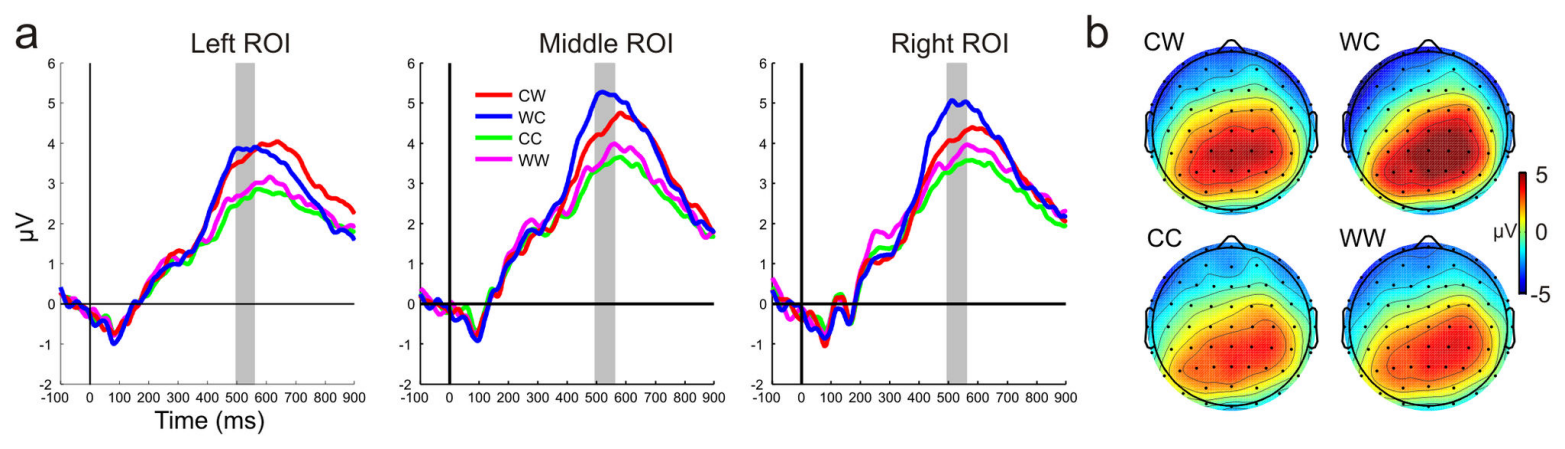
P3b responses. (a) ERPs averaged within the ROIs selected for P3b analysis the 500-560 ms range. Gray shadings indicate the 500-560 ms intervals where significant differences were found by ANOVA. Electrodes CP1 and P1 were selected for the left, CPz and Pz for the midline and CP2 and P2 for the right ROI. (b) Topographic maps of the P3b responses for the CW, WC, CC, and WW conditions within the 500-560 ms interval where the ANOVA yielded significant differences.

Figure 5 shows ERPs averaged within the ROIs and scalp topographies of the P3b potentials in the 500-560 ms interval to S2 stimuli. An ANOVA of amplitudes of S2 potentials in the 500-560 ms interval with factors of type (CC vs. WW vs. CW vs. WC) × laterality (left vs. middle vs. right) yielded a significant interaction [F(6,132) = 2.56, ε = 0.76, p <.05, η2 = 0.10]. Post-hoc Tukey HSD tests showed, that S2 of the ’different’ pairs (CW, WC) elicited larger positivity than the S2 stimuli of the ’same’ pairs (CC, WW). S2 responses to the ’same’ pairs (CC, WW) did not differ. Furthermore, potentials elicited by the second member of the WC pair were more positive than the second member of the CW pair at the middle and right ROIs. 

## Discussion

Participants’ behavioral data revealed the typical asymmetrical response to close-then-wide (CW) and wide-then-close (WC) pairs that is diagnostic of boundary extension (e.g., [4,7,15,34]). On CW trials the second view was rated as being closer to “same” than on WC trials. Participants were more confident of their responses on WC trials than CW trials, a finding that is consistent with the idea that the difference between the two views was more salient on WC trials. In the case of identical pairs (CC and WW), the response pattern diagnostic of boundary extension is for participants to rate the test picture as being more close-up than the original, signifying that the original is remembered with extended boundaries.

Typically, CC trials yield a greater boundary extension than do WW trials. In the current experiment, however, confirming our concerns about the large number of trials, this did not occur. Overall, the WW trials yielded boundary extension but the CC trials did not. However, when boundary ratings were analyzed for the first block of trials only (60 trials), both the CC and the WW trials each yielded significant boundary extension. Several factors may have contributed to the small BE ratings. Paradoxically, boundary extension tends to be greater under conditions typically associated with more accurate memory (small set sizes, distinctive stimuli and multisecond presentations) than those traditionally associated with memory errors [35]. To obtain ERP data, the number of trials was many times greater than is typically used in BE research. It may be that after viewing numerous similar pairings of close and wide single-object scenes, proactive interference may have affected participants’ responses on the rating scale (leading to an increase in “same” responses). Although the ratings were smaller than those typically observed in other BE studies, the critical response asymmetry to mismatched pairs was observed both in the first block of trials and across the entire experiment. Given this outcome, it is clear that large numbers of trials can be used in a BE experiment, but the results suggest that in future research additional measures should be taken to try to maximize BE, for example by presenting tighter close-up views and more distinctive scenes. This may help to minimize the potentially countervailing memory effects that can accumulate across large numbers of trials. Theoretically, sorting S1 trials according to whether the corresponding behavioral response indicated the presence of the BE effect [2] could have provided new insight about the time course of the scene extrapolation during the first stage of boundary extension. Unfortunately, however, the relatively low trial number where BE was present prevented us from analyzing ERPs to S1 scenes.

### Event-related potentials

ERP effects on CW and WC trials mirroring the behavioral response asymmetry were observed. We obtained two effects of this type, one in an earlier latency range (265-285 ms) and the other in a later (480-580 ms) latency range. The early effect differentiated CW from WC trials. We suggest that this difference reflects the fact that the test picture in the CW pairs more closed “matches” the first picture in the pair than on any of the other trials. In terms of the model presented in Figure 1, boundary extension in Stage 2 leads to greater similarity between the remembered view and the test picture. The late effect differentiated WC from the other three trial types. This difference may reflect the size of the “mismatch”, which is greater on WC trials than on the other three types of trials, because boundary extension in memory for the stimulus would increase the perceived difference between the now (even more expansive) wide-angle view and the close-up view in the test picture.

In the 265-285 ms range over the left temporal-parietal locations the second member of the CW pair elicited less negative responses. This ERP result suggests that not only is boundary extension evident following a brief interruption to the stimulus [4,7] but that the impact of the error on evaluation of the test picture is present as early as 265 ms following the onset of the test picture. It is worthy of note here that the mass univariate statistical approach we applied to take this first exploratory look at brain activity related to potential CW vs WC differences is a rather conservative method [28]. Although it allowed us to search for BE effects at many timepoints and at several electrodes, we cannot exclude the possibility that some BE effects may have emerged earlier than 265 ms which would have been revealed by conventional statistical tests (e.g., ANOVA). Specifically, visual inspection of the CW vs WC difference waveforms suggested the presence of a BE effect at ~160 ms poststimulus, however the mass univariate tests failed to reveal a statistically significant effect in this time range. It is worth mentioning here that analysis of response time of camera angle judgments showed no difference between conditions. Furthermore, mean response time in each of the four conditions was higher than 1450 ms, therefore it is unlikely that response-related activity have contributed to the observed ERP effects.

Major ERP peaks and troughs (P1, N1, P2) presumably reflect the activity of numerous independent sources summed by volume conduction of the brain [36]. Therefore although the time interval of the first BE effect coincided with the P2 component, the observed effect probably reflects the activity of an independent generator which contributes partially to the shape of the ERP waveforms as it was measured on the scalp. Nevertheless, as Figure 3 shows, the 265-285 ms corresponds to the falling part of the centro-parietal P2. The centro-parietal topography of P2 and the topography of the difference potential may reflect processes similar to the fMRI findings [5], in which attenuation of the neural response occurred on CW trials but not WC trials in RSC (thought to underlie navigation and placement of views within larger spatial contexts [37,38]) and in PPA (thought to be sensitive to the perception of a scene’s layout [19]). An activity difference in the present study emerged at electrodes over the centro-parietal regions, but over the occipital regions we obtained no such difference. Similarly, Park et al. [5] observed no boundary extension-related activity in the lateral occipital regions (which are thought to underlie object recognition). While it is difficult to localize ERP effects like the CW vs. CC, WW, WC difference of the present study, responses of striate and prestriate sources emerge over the occipital and/or parieto-occipital locations. In this respect the results of the two studies are similar.

So far no studies with concurrent event-related potential recording have compared stimuli that differ only in terms of slight changes in the scope of the view. Due to the posterior distribution, the late positivity of the present task is considered as a P3b (e.g., [39,40]). In the present study P3b to the second members of the stimulus pairs (S2) was larger in the ‘different’ (CW and WC) pairs compared to the ‘same’ (CC and WW) pairs. The smaller P3b of the ‘same’ pairs can be explained as a probability effect. It is well known, that stimulus probability affects P3b amplitude (e.g., [41]). We suggest that participants used three response categories in evaluating the S2 stimuli: closer, same and wider. Half of the trials were ‘same’, and the higher probability of this category resulted in smaller P3b amplitude.

Furthermore, the P3b component was larger in the WC than in the CW pairs. In the context of boundary extension, the WC vs. CW amplitude difference is more relevant, than the difference between the ‘same’ (CC and WW) pairs and the ‘different’ (CW and WC) pairs. A considerable body of research shows that larger demand for attention resources is accompanied by smaller P3b amplitude (e.g., [42,43]; see 44 for a review). Although the functional significance of the P3b component is debated (e.g., [45,46,47]), it is apparent that it peaks after the termination of stimulus evaluation processes. As the data show, in the WC condition participants were more confident in their judgments than in the other conditions. The larger uncertainty in the CW condition may result in smaller P3b amplitude. The lower attentional demand of the WC condition can be connected to the BE effect. The close-up stimulus in the WC pairs is more different from the memory representation of the first stimulus, than the wide angle stimulus in the CW pair. As results of early studies on late positivity show, the smaller the difference between the to-be-discredited stimuli, the smaller the amplitude of the late positivity (see 48 as a summary). Accordingly, in the present study P3b difference can be considered as a correlate of the boundary extension.

The results of the present ERP study show that not only does the misattribution of predicted information to vision leading to boundary extension occur rapidly, but also that the comparison of the memory representations of two views is rapid, emerging at about 265 ms after onset of the test picture. However, it is too early to come to a conclusion regarding the origin of the early CW vs. WC difference. It is possible that the differential activity is either the correlate of the detection of the relatively close ‘match’ following the wide test stimulus, or alternatively it signifies the detection of a relatively large ‘mismatch’ following the close test stimulus. However, in the light of predictive coding frameworks of perception [49,50], the latter alternative seems to be more plausible. This is also supported by the fact, that in this study half of the stimulus pairs were identical (CC, WW), and one quarter of the stimuli (CW pairs) were biased towards “sameness”, in a sense that the distorted (extended) memory of the C stimuli probably matched the W test stimulus better, than the distorted memory of W stimuli matched the C test stimuli. That is, 75% of the extended memory representations of the stimulus pairs were biased to a better match than on the WC trials representing 25% of the pairs.

According to this interpretation, “sameness” was the frequently presented category, which might have induced an expectation. If it was indeed the case, then the observed early ERP difference might index a ‘mismatch’ process, i.e., prediction error signals to WC test stimuli violating the “sameness” expectation. This is also in line with the result that stimuli with lower (subjective) probability elicited larger responses, i.e., the second member of the WC pairs evoked the largest P3b component. We suggest that future studies should investigate boundary extension-related brain responses in experiments where automatic predictive processes can be manipulated.

In the current paradigm the figure changes when the object is closer to or farther away from the camera, therefore the difference between objects wider from and closer to the camera is between smaller and larger objects. One might argue, that the observed effect might be explained by the lower task demands for changing focus from larger to smaller (WC) than for smaller to larger (CW). It has been shown that it takes less effort to narrow the focus of attention from a larger view on the object to a smaller one than to extend the focus of attention from a smaller view on the object to a larger one [51]. However, such an effect was shown with stimuli of large letters made up of a lot of smaller letters, where the ratio of global (larger) vs. local (smaller) letter size was about 6/1, whereas in our current study the average ratio between larger and smaller versions of the same object was about 1.1/1, therefore it is highly unlikely that differences in effort required to change attention from larger to smaller and smaller to larger could explain our findings.

## Summary

In summary, we observed behavioral and ERP correlates of BE in our study. We exploited a diagnostic response asymmetry to mismatched views (a closer and wider view of the same scene) in which the same pair of views is rated as more similar when the closer item is shown first than vice versa. Boundary ratings replicated the typical asymmetry. The behavioral results indicate that BE can be studied using a paradigm with a relatively large number of trials, although it is likely to weaken BE by introducing other types of memory errors that interact with detection of BE (e.g., [26]). We found an asymmetry in ERP responses in the 265-285 ms interval matching the behavioral results where the second member of the close-then-wide pairs evoked less negative responses at left parieto-temporal sites compared to the wide-then-close condition. This suggests the falsely remembered region beyond the view-boundaries of S1 is rapidly available and impacts assessment of the test picture within the first 265 ms of viewing. This observation adds to our current understanding of the early time course of BE-related error. Behavioral data showed that BE occurs across an interruption to sensory input that is commensurate with a saccade (e.g., 42 ms [7]). The ERP data observed in the current study demonstrates that the impact of BE on assessment of the second picture occurs at least as soon as the first 265 ms of viewing. This suggests that extrapolated scene structure may be computed rapidly enough to play a role in the integration of successive views during visual scanning.
